# Health Policy and Privacy Challenges Associated With Digital Technology

**DOI:** 10.1001/jamanetworkopen.2020.8285

**Published:** 2020-07-09

**Authors:** David Grande, Xochitl Luna Marti, Rachel Feuerstein-Simon, Raina M. Merchant, David A. Asch, Ashley Lewson, Carolyn C. Cannuscio

**Affiliations:** 1 Leonard Davis Institute of Health Economics, University of Pennsylvania, Philadelphia; 2 Perelman School of Medicine, Division of General Internal Medicine, University of Pennsylvania, Philadelphia; 3 Center for Public Health Initiatives, University of Pennsylvania, Philadelphia; 4 Perelman School of Medicine, Department of Emergency Medicine, University of Pennsylvania, Philadelphia; 5 Penn Medicine Center for Health Care Innovation, Philadelphia, Pennsylvania; 6 Department of Psychology, Indiana University–Purdue University Indianapolis, Indianapolis; 7 Perelman School of Medicine, Department of Family Medicine and Community Health, University of Pennsylvania, Philadelphia

## Abstract

**Importance:**

Digital technology is part of everyday life. Digital interactions generate large amounts of data that can reveal information about the health of individual consumers (the digital health footprint).

**Objective:**

Τo describe health privacy challenges associated with digital technology.

**Design, Setting, and Participants:**

For this qualitative study, In-depth, semistructured, qualitative interviews were conducted with 26 key experts from diverse fields in the US between January 1 and July 31, 2018. Open-ended questions and hypothetical scenarios were used to identify sources of digital information that contribute to consumers’ health-relevant digital footprints and challenges for health privacy. Participants also completed a survey instrument on which they rated the health relatedness of digital data sources.

**Main Outcomes and Measures:**

Health policy challenges associated with digital technology based on qualitative responses to expert interviews.

**Results:**

Although experts’ ratings of digital data sources suggested a possible distinction between health and nonhealth data, qualitative interviews uniformly indicated that all data can be health data, particularly when aggregated across sources and time. Five key characteristics of the digital health footprint were associated with health privacy policy challenges: invisibility (people are unaware of how their data are tracked), inaccuracy (data in the digital health footprint can be inaccurate), immortality (data have no expiration date and are aggregated over time), marketability (data have immense commercial value and are frequently bought and sold), and identifiability (individuals can be readily reidentified and anonymity is nearly impossible to achieve). There are virtually no regulatory structures in the US to protect health privacy in the context of the digital health footprint.

**Conclusions and Relevance:**

The findings suggest that a sector-specific approach to digital technology privacy in the US may be associated with inadequate health privacy protections.

## Key Points

**Question:**

What challenges for health privacy are associated with digital technology?

**Findings:**

In this qualitative study, 5 key challenges for health privacy were associated with digital technology: invisibility (people unaware of how they are tracked), inaccuracy (flawed data), immortality (data never expire), marketability (data are frequently bought and sold), and identifiability (individuals can be readily reidentified).

**Meaning:**

The findings suggest that a sector-specific approach to digital technology privacy in the US may be associated with inadequate health privacy protections.

## Teaser

This qualitative study assesses the association between digital technology and emerging challenges for health policy and privacy.

## Introduction

By June 2019, there were more than 4.4 billion internet users, an 83% increase in 5 years.^1^ More than half of the global population uses email.^2^ Seventy-three percent of Americans frequently access their bank accounts online.^3^ Every minute on Facebook, 510 000 comments are posted, 293 000 statuses are updated, and 136 000 photographs are uploaded.^4^ Digital interactions are obligatory, with central roles at home and work, resulting in a recorded stream of personal information.

Digital interactions, using mobile applications, searching the Internet, wearing connected devices, or conversing on social media, often generate health-relevant information. Smart watches and smartphone applications are in widespread use for tracking physical activity, fertility, and blood glucose levels.^5^ One step removed, data scientists have been able to identify chronic disease risk or depressed mood based on Internet searches and social media posts.^6,7,8,9,10^ Cars now record whether their drivers have gained or lost weight or strayed from their lanes.^11^ We refer to the sum of these health-relevant data as an individual’s *digital health footprint*.

Although the European Union implemented broad new consumer digital privacy regulations in 2018, the US has not adopted a comprehensive regulatory approach.^12^ Instead, the US has taken a sector-specific approach,^13^ with differential protections conferred on health care encounter data through the Health Insurance Portability and Accountability Act (HIPAA).^14^ In addition, genetic information, thought to be particularly sensitive, receives protections under the Genetic Information Nondiscrimination Act (GINA).^15^ These regulations leave wide swaths of digital consumer privacy unregulated.

Some privacy risks are attributable to illegal hacking. Those risks are managed by security systems and law enforcement. However, many of the challenges to health privacy are associated with data practices that are currently legal. We explored the privacy challenges associated with the digital health footprint through interviews with multidisciplinary experts. Those interviews informed a framework for considering the genesis, transformation, and application of the digital health footprint as well as challenging characteristics of the digital health footprint that may require policy attention.

## Methods

### Participants

For this qualitative study, we conducted interviews between January 1 and July 31, 2018, using purposive and convenience sampling to recruit 26 participants with diverse expertise in emerging digital technology and applications to health care and research. Specific areas of expertise included applications of digital technology to health (n = 12), data analytics and data mining (n = 12), health care innovation and business (n = 9), consumer behavior and preferences (n = 9), marketing (n = 7), health policy (n = 7), computer science (n = 7), privacy law (n = 4), ethics (n = 3), data security (n = 3), consumer advocacy (n = 3), and machine learning (n = 3) (categories not mutually exclusive). Experts were drawn from a range of sources, including national and international privacy committees and commissions and related research publications, and through convenience strategies beginning with our project advisory committee. Interview participants were compensated with $200. This study was reviewed and declared exempt by the institutional review board at the University of Pennsylvania. We were granted a waiver of written informed consent but obtained verbal informed consent from participants before their interview. All data were deidentified. This study followed the Standards for Reporting Qualitative Research (SRQR) reporting guideline.

### Design

Interviews were conducted using in-depth, semistructured, qualitative methods. The interview guide (eAppendix in the Supplement) was informed by a consequential ethics framework in which the presence or absence of a substantial risk of harm associated with a loss of privacy determines the need for protections.^16,17^ Through open-ended questions and hypothetical scenarios, we asked experts to identify current and emerging sources of digital information from outside health care that contribute to consumers’ health-relevant digital footprints. We also asked them to describe current and potential future applications of that information, anticipate potential harms and benefits, and consider approaches to addressing privacy concerns. The 20- to 60-minute interviews were audio-recorded and conducted over the telephone or in person by a trained research coordinator (A.L.). After each interview, a web-based follow-up questionnaire was sent to the participant. The questionnaire included a list of data sources, and experts were asked to rate the health relatedness, potential harm (ie, if disclosed), and potential benefit (ie, to individuals or society) on a scale of 0 to 100, with 0 being the least and 100 being the most. Interviews were recorded, transcribed by a professional transcription service, and deidentified before being uploaded to NVivo, version 12 (QSR International) for analysis.^18^

### Statistical Analysis

The study team developed a codebook through line-by-line, iterative reading and notation of transcripts, which produced 12 key categories.^19^ Two research coordinators (X.L.M., A.L.) trained in qualitative data analysis used the codebook to complete coding. To establish agreement, 14 interview transcripts were double coded. Interrater reliability was measured using percent agreement (96.5%) and the Cohen κ (0.68). After agreement was established, the researchers individually coded the remaining transcripts, which were then summarized in memos that were reviewed and discussed by the study team to identify patterns and synthesize cross-cutting themes. The results are reported thematically, with supporting quotes, to distill the most salient challenges for health policy.

## Results

A total of 26 experts were interviewed. The interviews informed a conceptual framework for understanding the digital health footprint and identifying potential leverage points for regulation or policy action. They also revealed 3 key themes: (1) the digital ecosystem offers no clear distinction between health and nonhealth information, (2) key characteristics of the digital footprint merit policy attention, and (3) few regulatory structures currently protect consumer privacy.

### Conceptual Framework

**Figure 1.**
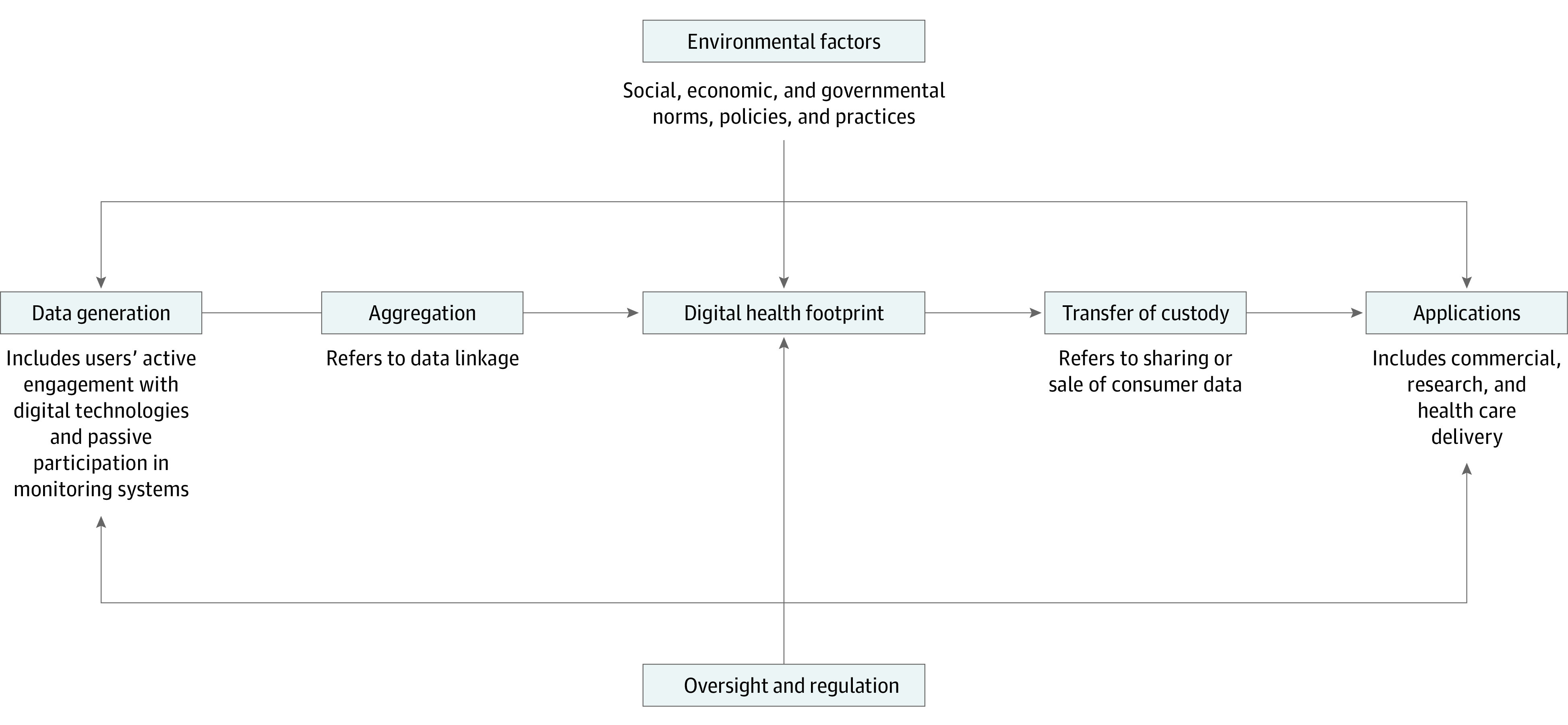
Findings From Interviews With Key Experts This figure shows the steps leading to the creation of the digital health footprint (including data generation and aggregation) and subsequent applications of digital data by a range of users across different sectors. This entire continuum is affected by overarching environmental factors that are associated with the collection and use of digital health data and the absence and/or presence of oversight and regulation.

Consumers’ everyday activities generate the digital health footprint, which is routinely aggregated, transferred (or commodified), and applied in a range of settings, including health care, business, and research. Figure 1 synthesizes the dynamic formation and transformation of the digital health footprint, as described by experts in this study.

Within the digital ecosystem, social, economic, and governmental norms, practices, and policies are the main contributors to the increasing reliance on digital technologies for core life tasks, including pragmatic economic transactions (eg, banking), governmental functions (eg, tax filing), and social exchanges (eg, texting). Together, these data-generating activities contribute to the digital health footprint, which is a person-specific, dynamically evolving collection of digital information that can be used to infer current health states or estimate future health states. Consumer data are aggregated or linked across platforms, allowing for more nuanced inferences about health than can be derived from any single data source.

Experts projected that, with the potential for improved estimation of health states, the digital health footprint will become more commercially valuable (eg, as health care systems increasingly rely on predictive analytics to manage patient care). Digital health footprints are being transferred from their original custodians to new commercial (and other) entities for applications unrelated to the original purpose of the data collected. Experts emphasized that the regulatory landscape attends almost exclusively to the ethical and legal concerns arising from electronic health records and genetic testing. However, wide-ranging information originating beyond the protected domains of health care and genetic testing contributes to the digital health footprint, with limited regulatory oversight or agreement regarding best practices.

### Distinction Between Health and Nonhealth Information

**Figure 2.**
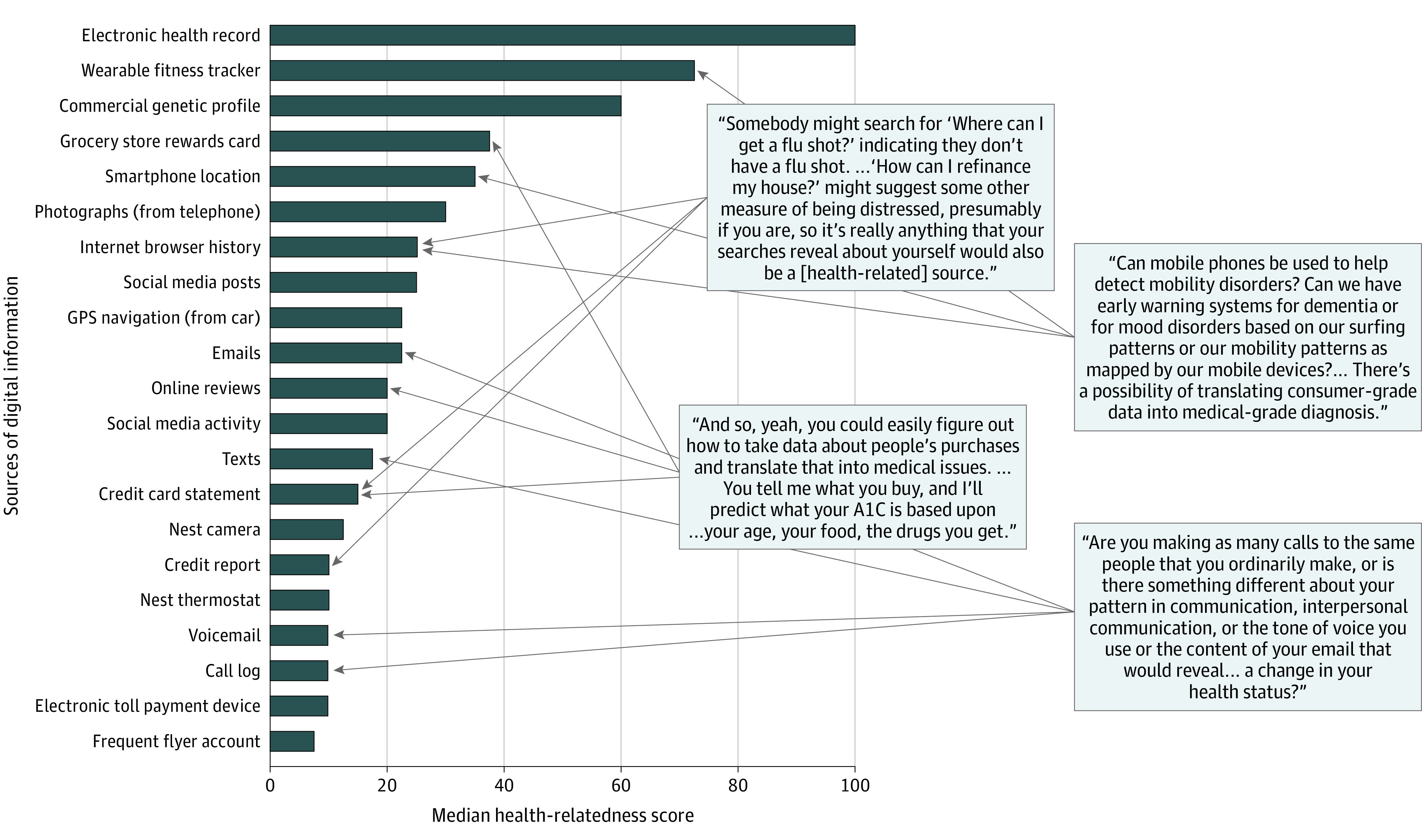
Responses of Experts Regarding the Health Relatedness of Digital Data Sources Experts were asked to rate the health relatedness of sources of digital data on a scale of 0 to 100, with 0 being the least and 100 being the most. These results are presented with quotes that demonstrate that interviewed experts were not able to draw distinctions between health and nonhealth data. A1C indicates glycated hemoglobin; GPS, Global Positioning System.

Experts rated distinct information streams that contribute to the digital health footprint (Figure 2) on a scale from 0 (not at all health related) to 100 (highly health related). They assigned highly variable scores to different information streams, with high scores for the electronic health record (median score, 100; IQR, 85-100), followed by fitness trackers (median score, 72.5; IQR, 52.5-80.0). Lower health-relatedness scores were assigned to commercial genetic profiles (median score, 60; IQR, 50-75), toll-tracking devices (median score, 10; IQR, 5-20), and frequent flyer accounts (median score, 7.50; IQR, 2.75-10.00).

The experts uniformly indicated that there are no clear distinctions between health-related and non–health-related data (Figure 2) and that all data can become health data. They noted that data are routinely aggregated across domains and over time, allowing for additional predictive analytics and increasingly precise characterization of health or risks. As one expert noted, “We’re moving from a time where health was measured directly using clinical measures to a new era where health is measured indirectly using...all the available information we leak on a daily basis.”

Experts summarized that “the line between just general digital data and health data is going to become so blurred...and the regulations aren’t going to catch up,” potentially introducing the risk of “discrimination based off of just 1 or 2 streams of information.”

### Key Characteristics of the Digital Health Footprint 

The experts identified 5 characteristics of the digital health footprint that may be associated with threats to consumer privacy (Table). The first characteristic was invisibility. An expert noted, “It would be very, very odd if someone followed you around…making notes of everywhere you went...and how much money you spent and what you saw and who you interacted with...We would call it stalking, right? But in the digital world, that’s just common behavior.”

**Table.** Key Characteristics of the Digital Health Footprint

Theme	Quotes	No. (%) of respondents (N = 26)
Invisibility	“One of the problems has been the use of these kinds of data in conjunction with other data sets. And so, one of the precautions is when a consumer agrees to share their particular data, not having—having the ability to control that the data doesn’t get combined with other information about them. So taken by itself, the idea that I might buy chocolate isn’t such a bad data point. Combining that with other things may point a particular kind of risk factors that I don’t want other people knowing or understanding about me.” “So that’s kind of like when people get a little bit of the tip of the iceberg and it freaks them out. And behind that little pair of shoes following you around is this huge infrastructure, really, like this kind of hidden web that’s bigger than the web you see, in a sense, and that is where all that information is getting collected....” “Well, part of it is that it’s very opaque, right? Companies who own this data are not necessarily revealing how they’re using it.”	22 (85)
Inaccuracy	“A close friend of mine had a type of cancer, and so I began doing some online searches that were focused on finding a physician to treat this person...about two weeks later, I’m on a national newspaper site, and I get an ad for a physician for treating this particular type of cancer pop up on the right-hand side of my page on the newspaper. It’s like ick, like ick, because they have targeted that based on my searches.” “We know that actually the data collected through these kind of devices is notoriously patchy in terms of its—well, certainly in terms of its accuracy and also in terms of its completeness, so I think findings taken from these kinds of data is—always needs to be taken with a pinch of salt.”	7 (27)
Immortality	“I think the way the systems are set up now, they allow for infinite selling and reuse and storage of this data for the targeted advertising.” “So how long does this stuff get kept? And the answer is usually forever because hard drives that they store it on are really cheap. So we store it forever. I kinda think whatever problems that we may have with these types of information being collected, you kind of exponentially get worse and they exponentially accrue. So if there’s data leaks or something, let’s say, some corporation has a list of everybody’s medical interests.”	8 (31)
Marketability	“I think the bigger impact is around the kind of political economy of these devices and the data which is generated, in that we have become creators of potentially valuable data for corporations in most cases.” “How many times do you and I use Google all day long? A lot—10, 20, 30, 40 times. And it’s free because you and I are the product. We are what’s being sold... And I think most people don’t really kind of exactly get that.”	14 (54)
Identifiability	“Once data leaves that first party and starts to get matched up with other datasets where you’re highly identifiable, then, now, you get—people often build these databases that are really, really, really powerful. So they know not only like, okay. Here’s all your ads, here’s are all your social behavior, but here’s your actual financial information or here’s your medical information. And that becomes very tricky because then people build full models of who you are.” “Well, it turned out [Strava] exposes the location of secret military bases in Afghanistan because it turns out military people like to do quantified self-stuff and didn’t realize that their data was gonna reveal the precise patterns of their patrols. So they can literally be seen from space in the data. So those sorts of things are starting to happen and they are going to crest and accelerate as people realize that by connecting these things to the internet and that it becomes possible to assemble this data in ways that no one ever expected.” “Lots of people have done quite credible research using public data sets and identifying individuals in these, and I think that’s the problem here too. Even if you say, ‘oh we use all this data anonymously,’ by definition the power of combining all these data sets means you can identify people quite easily.”	9 (35)

A refrain was that consumers are largely unaware of how and where their data are being tracked, used, and sold. In addition, consumers are fundamentally denied the opportunity to opt out of passive data collection (eg, surveillance cameras and facial recognition). The invisibility of the digital health footprint may contribute to low levels of consumer vigilance, especially in the context of unwieldy privacy policies.

Another identified characteristic was inaccuracy. An expert stated, “If you don’t take that information seriously and you think, ‘Oh, it’s just some quiz,’ and maybe you just randomly answer some [joking] response, it might still stick with you.”

The experts cautioned that the digital health footprint can generate inaccurate inferences because machines are literal in their data interpretation. Thus, the digital health footprint may contain ambiguous information about health behaviors or the social determinants of health. For example, a location tracker could note a visit to a clinic with abortion services, which may incorrectly signal that the person had an abortion. A subset of inaccuracy is the inadvertent bystander effect in which data from neighbors, friends, and social network members may be used to infer a person’s own behaviors, which may or may not be concordant. Experts raised concern that consumers have limited control to correct inaccuracies in their digital records.

The third characteristic was immortality of data. An expert noted, “Say I build a wellness app and I ask you to fill out extensive surveys about yourself…It gets mildly popular and I get an acquisition off of it...the data is going to go into a data broker and get endlessly resold and segmented.”

Experts were concerned that an infinite lifetime for health-relevant digital data presents a high risk of potential misuse, exposure, or other breaches. A small risk, sustained during a long period, translates into a high risk of an adverse event. Experts cautioned that consumers should (but usually do not) have opportunities to review and destroy their own personal data, including data they perceive to be potentially damaging.

Marketability was a fourth characteristic identified through the expert interviews. One participant mentioned, “There’s a lot of questions about whether it’s right that companies are selling people’s individual consumer data and then the buyer of data turns it into profitable products and the consumer never benefits from that in any way.”

Experts underscored that consumers’ digital information holds potential for scientific and clinical advances as well as for commercial gain, with a low likelihood of compensation to the people whose digital data are being traded and sold. Experts highlighted that there are few, if any, safeguards against exploitation and no established mechanisms for compensating the individuals who contribute to advances derived from digital health footprints. Moreover, commercialization opportunities may further motivate data collection and development of new applications.

The last characteristic was identifiability. An expert noted, “Eighty-five percent of people can be re-identified based on 3 GPS points; my home, my office, my children’s school, narrow me down to a really small number of people that that could uniquely be.”

Experts indicated that individuals can easily be reidentified through the merging of data streams, thus undermining promises of confidentiality. Identifiability may be used as a tool for screening and identification of risk. For example, aggregated data and improved algorithms may identify problematic or dangerous behavior, such as suicidality, before a consumer (or their health care practitioner) is aware. However, identifiability may also allow for unwanted targeting, for example, efforts to shape consumer opinions and behavior (eg, Cambridge Analytica’s purchase of Facebook data to shape political opinions^20^) or discrimination (eg, hiring decisions or insurance pricing).

### Few Regulatory Structures for Consumer Privacy Protection

One expert commented, “We have a lot of work to do in the US. We don’t currently have an omnibus privacy protection law...we’ve also got a technology environment that has allowed for a relatively Wild West approach to the use and sharing and re-use of personal data.” The experts consistently described current regulatory protections as limited and sector specific. They additionally noted a reliance on corporate and other entities to self-monitor and protect consumers’ interests.

## Discussion

We identified 3 key findings. First, there are no clear distinctions between data that are and are not health related. Second, the digital health footprint is associated with enduring health privacy challenges that transcend specific technologies or applications. Third, the digital health footprint is largely unregulated. These findings may have implications for health privacy and policy.

Data scientists draw inferences about health from wide-ranging, routinely collected data.^21,22,23^ Facebook has assessed linguistic nuance to identify mental health problems,^24^ smart mattresses monitor sleep habits,^25^ and location tracking can identify individuals who visit abortion clinics.^26^ Beyond these focused applications, data brokers are now commodifying aggregated digital data to fuel predictive analytics that can be applied in different settings (eg, health risk scores).^27,28^ The enactment of HIPAA and GINA reflected regulators’ intent to confer special status on health information and therefore heightened consumer protections.^29,30,31,32^ A key finding from this study—that all data are health data—suggests that the privacy protections of HIPAA and GINA are inadequate and obsolete. The views of the experts who we interviewed are in line with an increasing body of academic and lay literature on the relevance of digital information to health.^14,33,34^ In the current digital landscape, a multisectoral regulatory approach is necessary to protect consumers’ health privacy.^13,35^

Several challenges of the digital health footprint may transcend evolving technologies, posing persistent questions for health policy. Policy lessons can be drawn from long-standing debates about genetic privacy because genetic data share these qualities.^36,37,38^

Ethicists have addressed the case of Henrietta Lacks, whose cervical cancer cells were culled at Johns Hopkins Hospital and transformed into an immortal cell line still used today to advance scientific research.^39^ Like Henrietta Lacks’ cells (HeLa cells), the digital health footprint is immortal, having no set expiration date and no clear way to destroy data, especially when the chain of custody is long and digital copies exist across multiple platforms. In addition, policy makers must contend with fundamental questions of data ownership, consent, and just compensation to the people whose personal data (genetic or digital) are being repurposed or sold.^40^

As with genetic information, the digital health footprint is highly person specific. Data scientists have demonstrated that, even without direct identifiers (eg, name and address), individuals can be identified readily in population databases (eg, location tracking) using a relatively small number of data points.^41,42,43^ Recognizing that HIPAA only protects the fraction of health information generated in health care encounters, new standards must be developed for the deidentification, use, and protection of information contained in the digital health footprint. In advancing new regulatory approaches, a key challenge is to balance consumer privacy and the limiting of potential harms with the potential for health care advances that may be derived from the digital health footprint.^44,45,46^

Breaches of health information privacy can lead to social stigma, embarrassment, and economic harm (eg, insurance discrimination).^47^ The digital health footprint expands the scope and scale of potential data breaches and raises additional concerns. For example, machine learning and algorithms have been shown to perpetuate rather than eliminate racial biases and discrimination. Benjamin^48^ has described this phenomenon as the New Jim Code, referring to the embedded, invisible, and potentially injurious bias in automated systems. In a recent study,^49^ a health system algorithm relied on health care utilization as a proxy for illness severity. At similar levels of illness complexity, black patients had used fewer health services than white patients; thus, the algorithm did not see how sick the black patients actually were. Therefore, the code systematically underidentified black patients in need of supportive health interventions.^49,50^

Even without a specific social or economic harm, ethicists and policy makers have made the case that if a consumer is forced to live in a society in which nothing is private, consumers will lose their sense of individual dignity,^16,17^ potentially eroding social trust or generating adverse psychological sequelae.^51^ Without trust, it is difficult for individuals to have meaningful relationships in personal and professional spheres of their lives with long-term social consequences.^52^

The US is notable because of its sector-specific regulatory approach, which shaped HIPAA as the approach to privacy in the health sector. Although specific policy solutions were beyond the scope of our interviews with experts, many pointed to the model of the European Union General Data Protection Regulation. The General Data Protection Regulation establishes individual privacy rights across sectors, codifying expanded transparency of data collection and use, increasing consumer control and data access, and limiting the immortality of data through provisions, such as the right to be forgotten.^53^

In the US, the California Consumer Privacy Act became law as of January 1, 2020, allowing residents to access personal information collected about them digitally and to opt out of the commercialization of their personal data.^54^ Extensive efforts are needed to understand how these protections will be interpreted, adopted, enforced, and replicated or expanded elsewhere in the US.

### Limitations

This study has limitations. First, this study is qualitative and therefore intended to identify the breadth of issues to consider around digital health privacy as opposed to quantifying the prevalence of views. The relatively small sample size for this qualitative approach means that our experts may not be representative of the broader population of experts in the respective fields we sampled. In addition, despite efforts to achieve a diverse sample, some perspectives (eg, employees of companies with proprietary interests) may be underrepresented. Second, the digital privacy landscape is rapidly evolving and the subject of intensive media focus.^55^ Results are reflective of the period (2018) during which the study was conducted. Third, our experts were not sampled to identify the full breadth of views regarding privacy law and potential policy solutions, and our interview guide did not seek to arrive at policy solutions. Fourth, social desirability also may have been operative.

## Conclusions

This study suggest that there is no distinction between health and nonhealth data. Far-reaching sources of data contribute to the digital health footprint, with implications for most, if not all, US individuals. The findings also suggest that the US should reconsider definitions of health privacy and develop appropriate safeguards as digital technology permeates nearly all aspects of everyday life.

## Notes

Supplement. **eAppendix.** Digital Health Privacy Expert Interview Guide
