# Supplementary material for: Health Policy and Privacy Challenges Associated With Digital Technology
Source: JAMA Netw Open. 2020 Jul 9;3(7):e208285. doi: 10.1001/jamanetworkopen.2020.8285 (PMC7348687; doi:10.1001/jamanetworkopen.2020.8285)
Supplement: Supplement. — eAppendix. Digital Health Privacy Expert Interview Guide [file jamanetwopen-3-e208285-s001.pdf]

Supplementary Online Content

Grande D, Luna Marti X, Feuerstein-Simon R, et al. Health policy and privacy challenges associated with digital technology. *JAMA Netw Open*. 2020;3(7):e208285. doi:10.1001/jamanetworkopen.2020.8285

eAppendix. Digital Health Privacy Expert Interview Guide

This supplementary material has been provided by the authors to give readers additional information about their work.

eAppendix. Digital Health Privacy Expert Interview Guide

Introduction:

Thank you for your interest in sharing your thoughts. I am part of a research team at the University of Pennsylvania. Our goal is to learn more about experts' perspective on the expanding role of consumers' digital information and the opportunities for innovation and resulting privacy challenges.

Our conversation should last no more than one hour. Please know that there are no right or wrong answers—we are interested in your thoughts and opinions.

I would like to begin by having you describe what you see as most important about digital behavior and the information consumers are leaving behind.

- Off the top of your head, please list a few kinds of information are people leaving behind online and when they use digital technology. Where and how are consumers generating their digital footprint?
- Please choose an example that you listed and tell me how that information is being used today
    - *Follow up for marketing examples*: What aspects of digital marketing differ from previous marketing techniques?
- What are the possible benefits/harms of using this information? *Alternate order*
- What protections if any are in place for consumers?
- In what ways are these protections sufficient/ insufficient? *Alternate order*

As you know, we're health researchers. We'd like to learn more about the ways in which consumers' digital footprints reveal something about their health. Which sources on your list reveal something about the health of consumers?

- What are some sources of digital data that directly reveal something about health and what are some that indirectly reveal something about health?
- How are the sources of this information changing over time?
- What are some of the most novel and innovative sources of information that could change the landscape in the next 2-4 years?

Before we move on to some scenarios, I'd like to learn more about what your top priorities are regarding consumers' digital health information

- What is the biggest challenge / opportunity? *Alternate order*
- How does consumers' digital health information differ from other digital information?
- Who should be responsible for managing this?
- As you know, HIPAA sets a national standard for health information privacy. Could you discuss the ways in which HIPAA has harmed or helped innovation?

© 2020 Grande D et al. *JAMA Network Open.*

Next, I would like to review several hypothetical scenarios for the use of consumer digital information for health-related purposes. For each example I am going to ask you to talk about whether you think it is a good idea or a bad idea and why you think so, as well as any necessary precautions. Let me start with the first example.

(Scenarios previously discussed at length above would be skipped)

Scenario 1

A health insurance company is trying to find ways to keep people healthier and save money. They have found that consumers that buy certain kinds of food are more likely to develop diabetes. The insurance company is planning a program where they will access the grocery shopping records of their patients from grocery stores. The health insurance company will use this information to find out who is at high risk of developing diabetes, then send those people tips and advice on how they can prevent diabetes by making changes to the food they buy.

- *What do you think about this idea?*
- *Positive response → what things do you like? What would make you not like it?*
- *Negative response → what things don't you like? What could make you like it?*
- *Are there any limits or protections you think are necessary?*
- *If your insurance company offered a program like this, would you want to participate? Why/why not?*

Scenario 2

A doctor's office is trying to find ways to prevent people from getting sick and needing to go to the hospital. They have found that patients that search on the internet for certain symptoms are more likely to get sick and need to go to an emergency room. This doctor's office is planning a new program where they will access internet searches of their patients and contact patients that search for certain symptoms to try to start treatment sooner.

- *What do you think about this idea?*
- *Positive response → what things do you like? What would make you not like it?*
- *Negative response → what things don't you like? What could make you like it?*
- *Are there any limits or protections you think are necessary?*
- *If your doctor offered a program like this, would you want to participate? Why/why not?*

Scenario 3

University researchers are trying to find ways to prevent cancer. Researchers at a nearby university hospital are starting a research study where they will track patients over time to try to determine causes of cancer. In addition to using medical records, the research team will use

© 2020 Grande D et al. *JAMA Network Open.*

location information from patient's smartphones so they can study how the places where people spend most of their time impact their risk of getting cancer. The researchers want to use this knowledge to help develop public health strategies in the future that could reduce the number of people with cancer.

- *What do you think about this idea?*
- *Positive response → what things do you like? What would make you not like it?*
- *Negative response → what things don't you like? What could make you like it?*
- *Are there any limits or protections you think are necessary?*
- *If a local university offered a program like this, would you want to participate? Why/why not?*

Scenario 4

DigiHealth is a company selling a new smartphone app that can automatically collect and store information on places users visit and the food they eat so that it can give advice on ways to lower their risk of obesity. The app tracks where users go using location services on their smartphone and tracks what they eat by having them upload a picture of their meals. DigiHealth is able to offer the App for free because it shares user information with advertisers so they can send out grocery coupons.

- *What do you think about this idea?*
- *Positive response → what things do you like? What would make you not like it?*
- *Negative response → what things don't you like? What could make you like it?*
- *Are there any limits or protections you think are necessary?*
- *If DigiHealth offered an App like this, would you want it?*

Is there anyone else we should speak to about this topic?

- Is there anyone with a knowledge of industry/ commercial applications that you'd suggest?
- If so, would you be willing to make an introduction?

© 2020 Grande D et al. *JAMA Network Open.*
